# A new 500 kb haplotype associated with high CD8+ T-lymphocyte numbers predicts a less severe expression of hereditary hemochromatosis

**DOI:** 10.1186/1471-2350-9-97

**Published:** 2008-11-06

**Authors:** Eugénia Cruz, Chris Whittington, Samuel H Krikler, Cláudia Mascarenhas, Rosa Lacerda, Jorge Vieira, Graça Porto

**Affiliations:** 1 Clinical Hematology, Santo António Hospital, Porto, Portugal; 2 Iron Genes and the Immune System (IRIS), IBMC-Instituto de Biologia Molecular e Celular, Universidade do Porto, Portugal; 3 Department of Family Practice, University of British Columbia, Vancouver, Canada; 4 Department of Pathology and Laboratory Medicine, University of British Columbia, Vancouver, Canada; 5 BC Biomedical Laboratories, Surrey, Canada; 6 Molecular Immunology and Pathology, ICBAS-Instituto de Ciências Biomédicas de Abel Salazar, Universidade do Porto, Portugal; 7 Molecular Evolution, IBMC-Instituto de Biologia Molecular e Celular, Universidade do Porto, Portugal

## Abstract

**Background:**

Hereditary Hemochromatosis(HH) is a common genetic disorder of iron overload where the large majority of patients are homozygous for one ancestral mutation in the *HFE *gene. In spite of this remarkable genetic homogeneity, the condition is clinically heterogeneous, varying from a severe disease to an asymptomatic phenotype with only abnormal biochemical parameters. The recent recognition of the variable penetrance of the HH mutation in different large population studies demands the need to search for new modifiers of its phenotypic expression. The present study follows previous observations that MHC class-I linked genetic markers, associated with the setting of CD8+ T-lymphocyte numbers, could be clinically relevant modifiers of the phenotypic expression in HH, and aimed to find new markers that could be used as more reliable prognostic variables.

**Methods:**

Haplotype analysis, including seven genetic markers within a 1 Mb region around the microsatellite D6S105 was performed in a group of 56 previously characterized C282Y homozygous Portuguese patients. Parameters analyzed in this study were total body iron stores, clinical manifestations related with HH and immunological parameters (total lymphocyte numbers, CD4+ and CD8+ T-lymphocyte numbers). An independent group of 10 C282Y homozygous patients from Vancouver, Canada, were also included in this study and analyzed for the same parameters.

**Results:**

A highly conserved ancestral haplotype defined by the SNP markers PGBD1-A, ZNF193-A, ZNF165-T (designated as A-A-T) was found associated with both abnormally low CD8+ T-lymphocyte numbers and the development of a severe clinical expression of HH. In a small proportion of patients, another conserved haplotype defined by the SNP markers PGBD1-G, ZNF193-G, ZNF165-G (designated as G-G-G) was found associated with high CD8+ T-lymphocyte numbers and a milder clinical expression. Remarkably, the two conserved haplotypes defined in Portuguese patients were also observed in the geographically different population of Canadian patients, also predicting CD8+ T-lymphocyte numbers and the severity of disease.

**Conclusion:**

These results may have important implications not only for approaching the question of the penetrance of the hemochromatosis gene in different world populations but also to further narrow the region of interest to find a candidate gene involved in the setting of CD8+ T-lymphocyte numbers in humans.

## Background

Hereditary hemochromatosis (HH) is characterized by an inappropriately high iron absorption causing progressive iron loading of parenchymal cells of the liver and other organs with consequent tissue damage and dysfunction, leading to potentially lethal clinical consequences such as diabetes, liver cirrhosis and hepatocarcinoma [1]. The great majority of HH patients are homozygous for the C282Y mutation in *HFE*, a non-classical MHC class-I gene involved in the regulation of iron metabolism [2]. In spite of this great genetic homogeneity, the clinical heterogeneity is variable. Some patients exhibit a clinically severe disease while many C282Y homozygotes are apparently healthy showing only abnormal biochemical parameters and nonspecific symptoms such as fatigue and arthralgia [3-6]. Although gender, age and environmental factors partially explain the variability observed in iron accumulation and associated clinical presentation, these are not sufficient to explain all the phenotypic heterogeneity observed in clinical practice [7,8]. Recently, the recognition of variable penetrance of the C282Y mutation in different large population screening studies [8-14] has strengthened the need to search for new clinically relevant modifiers of phenotypic expression including new genetic modifiers.

We have previously shown that a large proportion of HH patients have consistently low CD8+ T-lymphocyte numbers correlating with a more severe expression of iron overload [15-18]. Low total lymphocytes counts, reflective of low CD8+ T-cell counts, were also shown in HH patients from the north of Portugal [19] and from Alabama (United States) [20] and those numbers were inversely associated with the amount of iron removed by phlebotomies [19,20]. The CD8+ T-lymphocyte abnormality was shown to be genetically transmitted, associated with the inheritance of particular HLA haplotypes [21,22]. More recent evidence was provided that stable numbers of peripheral blood CD8+ T lymphocytes are partially determined by genetic factors located close to the microsatellite marker D6S105 at the MHC-class-I region, close to the *HFE *gene [22,23]. Importantly, this same genetic region had been proposed some years ago as a putative location for modifiers of iron overload in Australian HH patients [24]. It is therefore highly probable that a major genetic trait contributing to the CD8+ T-lymphocyte abnormalities in HH patients is inherited in particular haplotypes, in linkage disequilibrium with the C282Y mutation, and, directly or indirectly, may contribute to the heterogeneity in the clinical expression of HH.

With the objective of identifying a better marker predicting both the inheritance of CD8+ T-cell numbers and the severity of expression in HH, haplotype analysis (including seven genetic markers within a 1 megabase region around the microsatellite D6S105) was performed in a group of 56 previously characterized C282Y homozygous Portuguese patients. Two different conserved haplotypes, with 500 kilobases (Kb) approximately, were identified and correlated with the phenotypic and clinical variables. In order to extend the significance of the results found in the Portuguese patients to a geographically different population, an additional group of 10 patients from Vancouver, Canada, was tested for the same genetic markers.

## Methods

### Study population

Two different populations were analyzed in the present study. The first group included 56 HH subjects, all homozygous for the C282Y mutation of the *HFE *gene, identified between 1985 and 2007 and regularly followed up at the Hemochromatosis Outpatient Clinic of Santo António Hospital, Porto and Predictive and Preventive Genetic Centre, Porto. These subjects were all Caucasians from the north of Portugal and included 45 probands detected in the context of suggestive clinical picture of hemochromatosis, generally with related clinical manifestations, or detected accidentally after a routine test and generally asymptomatic. Twenty-seven were males with mean age at diagnosis 46 ± 12 years and 18 were females with mean age at diagnosis 47 ± 10 years. Eleven patients were family members detected in the context of systematic family screening programs. These were 4 males with mean age at diagnosis 42 ± 5 years and 7 females with mean age at diagnosis 43 ± 15 years.

With the objective of extending the results obtained in the Portuguese patients to a geographically distinct population, an additional group of 10 HH patients homozygous for the C282Y mutation followed up at Dr Whittington's practice in Abbotsford, near Vancouver, Canada, were included in this study. Two patients (siblings) were from Germany, three patients were from the Netherlands, one was of Irish extraction, one was of mixed English and North American Indian extraction, two were Mennonites and another was adopted with unknown heritage. Five were males (median age at diagnosis 48 ± 7 years) and 5 were females (median age at diagnosis 61 ± 16 years). Three patients were detected in the context of suggestive clinical picture of hemochromatosis and had clinical symptoms, two were detected in the context of nonspecific clinical symptoms such as arthralgia and fatigue, and three were detected in routine tests and had no symptoms. Finally, two patients were family members detected in the context of family screening programs.

The study was approved by the ethical committee of Santo António Hospital including an informed consent obtained from patients according to the Helsinki declaration.

### Clinical characterization of subjects

Clinical data from the patients included in the study were carefully reviewed from their clinical files by one dedicated physician for each group of patients. The clinical parameters used in the analysis included: biochemical parameters of iron metabolism (TfSat and serum ferritin) determined at diagnosis by standard techniques as described [19], total body iron stores (TBIS) determined by quantitative phlebotomies [25] and the presence of clinical manifestations related to HH.

Clinical evaluation of the group of C282Y homozygous Portuguese patients was previously described in detail elsewhere [19,26]. Twenty-five were symptomatic patients presenting with one or more of the following manifestations: liver cirrhosis/fibrosis, diabetes, arthropathy, hypopituitarism, skin pigmentation or cardiac abnormalities, and removed an average of 9.3 ± 4.7 g of iron (between 2.5 and 17.6 g) by intensive phlebotomies. Twenty-eight were asymptomatic patients presenting with only biochemical evidence of iron overload and removed an average of 4.8 ± 2.8 g of iron (between 1.1 and 10.8 g) by intensive phlebotomies. Three patients had associated immunological conditions (two were hepatitis B virus carriers and one had a chronic monoclonal expansion of CD8+ T cells) and were *a posteriori *excluded from clinical analysis.

The group of Canadian patients had a greater heterogeneity in terms of clinical presentation than the Portuguese patients. Three had associated immunological diseases (small cell B lymphoma, celiac disease and Hashimoto's thyroiditis) and one had a viral infectious disease (hepatitis C infection). In terms of clinical manifestations 5 were symptomatic patients that removed an average of 15.0 ± 11.4 g of iron (between 2.0 and 28.5 g) by phlebotomies and 5 were asymptomatic patients that removed an average of 2.38 ± 1.7 g of iron (between 0.5 and 5.0 g) by intensive treatment.

### Immunological characterization of subjects

The immunological characterization of patients included the number of peripheral blood total lymphocytes and their sub-populations T-CD8+ and T-CD4+. The T-lymphocyte subpopulations were determined by FACS analysis using anti-CD3, anti-CD4 and anti-CD8 monoclonal antibodies as previously described in detail [21]. For the purpose of phenotypic characterization of patients, total lymphocyte numbers, CD4+ T-lymphocyte numbers and CD8+ T-lymphocyte numbers were considered "low" when they were ≤ 2.12, ≤ 0.90 and ≤ 0.41 × 10^6^/ml, respectively, and were considered "high" when > 2.12, > 0.90 and > 0.41 × 10^6^/ml, respectively, as defined in previous studies of lymphocyte populations in hemochromatosis [21,22,26]. These cut-off values were based on the median values of the parameters previously established on a control population from the north of Portugal [21].

### Genetic characterization of HH subjects

All subjects (56 Portuguese and 10 Canadian) had been previously genotyped for *HFE *mutations (H63D and C282Y) and they are all homozygous for the C282Y mutation. Forty-seven Portuguese HH patients were previously genotyped for microsatellites D6S2222 and D6S105 [22] and this information was included in this study. Genetic data from the Portuguese patients have been partially published [22].

**Figure 1:**
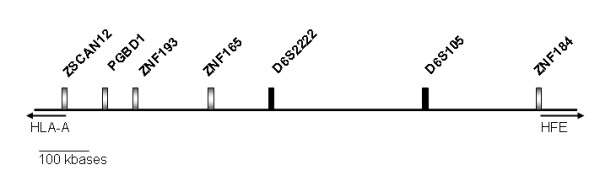
**Physical map of the genetic markers used in the present study and their relative location at scale.**

For the purpose of this study, 5 single nucleotide polymorphisms (SNPs) localized in the region around the microsatellite D6S105 in the 6p21.3 region were genotyped in all patients. These SNPs are localized in the following genes: zinc finger and SCAN domain containing 12 (ZSCAN12), piggyBac transposable element derived 1 (PGBD1), zinc finger protein (ZNF) 193, ZNF165 and ZNF184. Selection of SNPs was based on the location in the region and the frequency of alleles. These markers define a region of 1 megabase (Mb) (Figure 1) centromeric to *HFE *approximately 1.4 Mb and telomeric to HLA-A approximately 1.5 Mb.

SNP genotyping was performed by gene sequencing. Briefly, genomic DNA (gDNA) was extracted from peripheral blood or stored. gDNA and amplicons containing the selected loci were PCR-amplified using specific primers. Amplicons were then electrophoresed and extracted from the gel with the QIAquick Gel Extraction Kit (Quiagen). Sequencing reactions were prepared with the Big Dye Terminator v1.1 Cycle Sequencing kit (Applied Biosystems) and loaded in an ABI prism 310 Genetic Analyzer Sequencer (Applied Biosystems).

### Haplotype definition

For the purpose of this study, extended haplotypes were inferred using the program PHASE , as described previously in Vieira et al. 2007 [23]. Extended haplotypes were defined with the information on the genotype of 7 markers: ZSCAN12, PGBD1, ZNF193, ZNF165, D6S2222, D6S105 and ZNF184 (Figure 1). The phase of length polymorphisms at microsatellite markers D6S105 and D6S2222 was known from family studies (data not shown), and this information was used when running PHASE. In few cases (n = 9), information was missing for some individuals at some of the markers scored. In those cases, missing alleles were inferred using PHASE. A total of 14 different extended haplotypes (1 Mb) were defined in the group of 56 Portuguese HH subjects and their frequencies estimated in the population (Table 1). More restricted haplotypes (500 Kb) were further defined according to the pattern of allele conservation (conserved haplotypes).

**Table 1:** Inferred haplotypes present in a sample of 56 Portuguese HH patients

**#**	**ZSCAN12**	**PGBD1**	**ZNF193**	**ZNF 165**	**D6S2222**	**D6S105**	**ZNF184**	**N**	**Frequency**
1	**A**	**A**	**A**	**T**	**247**	**150**	**G**	82	73.2%
2	G	**A**	**A**	**T**	**247**	**150**	**G**	8	7.1%
3	**A**	**A**	**A**	**T**	249	150	G	5	4.5%
4	**A**	**A**	**A**	**T**	**247**	160	G	1	0.9%
5	**A**	**A**	**A**	**T**	**247**	148	G	1	0.9%
6	**A**	**A**	**A**	**T**	249	158	G	1	0.9%
7	**A**	**A**	**A**	**T**	n.a.	170	G	1	0.9%
8	G	**A**	**A**	**T**	**247**	148	G	1	0.9%
9	**A**	**A**	**A**	**T**	**247**	**150**	T	1	0.9%
10	**A**	**A**	**A**	**T**	245	**150**	T	2	1.8%
11	G	**A**	**A**	**T**	**247**	**150**	T	2	1.8%

12	**G**	**G**	**G**	**G**	**249**	150	G	4	3.6%
13	**G**	**G**	**G**	**G**	**249**	160	G	2	1.8%

14	G	A	G	G	249	150	T	1	0.9%

Alleles in bold represent conserved haplotype regions.

A: adenine; G: guanine; T: thymine. For microsatellite markers the size of the amplified PCR product is given (in base pairs).

n.a.: not available

For the purpose of defining the haplotypes in the 10 Canadian HH patients the PHASE program was run with the information on the genotype of 5 SNP markers (ZSCAN12, PGBD1, ZNF193, ZNF165 and ZNF184) determined in this population. The haplotypes found were the same as described in the Portuguese HH patients.

### Statistical analysis

Association studies between T-lymphocyte subpopulations and the genetic markers were performed in the Portuguese HH patients. Association studies were first performed on a single locus basis (for the seven markers included in the extended haplotypes) followed by analysis of the more restricted conserved haplotypes (see above). Moreover, since each subject carries two haplotypes, a third set of analyses was performed considering the combination of the two inherited conserved haplotypes. Finally, the combination of conserved haplotypes was used to analyze its impact on the clinical expression of the disease, both in terms of the amount of iron mobilized by phlebotomies (TBIS) and the clinical manifestations. In order to validate the results obtained with the analysis of the Portuguese HH patients, the impact of the combined haplotypes on both the number of CD8+ T cells and the phenotypic expression of HH was further analyzed including the whole group of patients (Portuguese and Canadian).

For the statistical analyses that include CD8+ T lymphocytes, patients with clinical conditions known to influence those numbers (such as autoimmune or viral diseases) were excluded (three Portuguese patients, two carriers of hepatitis B virus and one with chronic monoclonal expansion of CD8+ T cells, and 4 Canadian patients with: hepatitis C infection, small cell B lymphoma, celiac disease and Hashimoto's thyroiditis).

Group means were compared by the Student T-test when two groups were analyzed or by one-way analysis of variance (ANOVA) when more than two groups were analyzed. The Chi-square test was used to test the fitness of data to the normal distribution. Independence between categorical data was tested using the Chi-Square test. The Yates correction was used when small samples (< 5) were tested. All statistical tests were performed at 0.05 level of significance and all *p *values are two-sided. Data were analyzed by Statgraphics software (Statgraphics Graphics System, version 7.0).

## Results

### 1. Definition of haplotypes in a 1 Mb region around the microsatellite D6S105 in Portuguese HH subjects

For the characterization of a 1 Mb region in haplotypes carrying the C282Y mutation, information on the genotype of 7 genetic markers, ZSCAN12, PGBD1, ZNF193, ZNF165, D6S2222, D6S105 and ZNF184, was used (Figure 1). A total of 112 extended haplotypes were defined in 56 Portuguese HH subjects (see Material and Methods). Haplotypes were aligned according to the similarity to the most common haplotype. Results are shown in Table 1. The predominant extended haplotype was found in 73% (82/112) of chromosomes and is defined by: ZSCAN12-A, PGBD1-A, ZNF193-A, ZNF165-T, D6S2222-247, D6S105-150, ZNF184-G (haplotype #1, Table 1). The high frequency of this haplotype suggests that it is the ancestral haplotype in the evolutionary history of the C282Y mutation in this population. Several extended haplotypes differing from the ancestral in only 1 or 2 markers are found with frequencies of 0.9 to 7.1% (haplotypes #2 to #11, Table 1). All these extended haplotypes maintain a highly conserved region of approximately 500 Kb defined by PGBD1-A; ZNF193-A; ZNF165-T (see Table 1).

Only 3 extended haplotypes differing from the ancestral in more than 5 markers were found in this population of patients. Two of them (haplotypes #12 and #13) have a new conserved region defined by: ZSCAN12-G, PGBD1-G, ZNF193-G, ZNF165-G, D6S2222-249 and were found with a global frequency of 5.4% (6/112).

### 2. Association of CD8+ T-lymphocyte numbers with genetic markers in the region around the microsatellite D6S105 in Portuguese HH subjects

#### 2.1. Single locus analysis

For the purpose of investigating the influence of the region under study on CD8+ T-lymphocyte numbers, association studies were first performed between CD8+ T-lymphocyte numbers and the alleles of each genetic marker by one-way ANOVA. Allele diversity in this group of HH patients is evident for the markers ZSCAN12, D6S2222, D6S105 and ZNF184 (see Table 1). No association was found between any of these individual markers and the number of CD8+ T lymphocytes (data not shown). In contrast, a statistically significant association was found between CD8+ T-lymphocyte numbers and each of the SNP markers PGBD1 (p = 0.0109), ZNF193 (p = 0.027) and ZNF165 (p = 0.027). The alleles PGBD1-A, ZNF193-A and ZNF165-T were associated with "low" CD8+ T-cell counts (0.37 ± 0.17, 0.37 ± 0.17 and 0.37 ± 0.17 × 10^6^/ml, respectively) while the alleles PGBD1-G, ZNF193-G and ZNF165-G were associated with "high" CD8+ T-cell counts (0.55 ± 0.14, 0.51 ± 0.15 and 0.51 ± 0.15 × 10^6^/ml, respectively).

A statistically significant result was observed when associations of total lymphocyte counts and each of the SNP markers PGBD1 (p = 0.008), ZNF193 (p = 0.038) and ZNF165 (p = 0.038) were tested. The alleles PGBD1-A, ZNF193-A and ZNF165-T were associated with "low" total lymphocyte counts (2.00 ± 0.58, 2.01 ± 0.58 and 2.01 ± 0.58 × 10^6^/ml, respectively) while the alleles PGBD1-G, ZNF193-G and ZNF165-G were associated with "high" total lymphocyte counts (2.65 ± 0.42, 2.48 ± 0.58 and 2.48 ± 0.58 × 10^6^/ml, respectively). No statistically significant associations were found for total lymphocyte counts and any of the other markers used (data not shown). No statistically significant effect was found on the numbers of CD4 + T cells with any of the genetic markers tested (data not shown).

#### 2.2. Analyses of conserved haplotypes

It is relevant to note that the 3 alleles associated with "low" CD8+ T-cell numbers are found in linkage disequilibrium in the ancestral haplotype (PGBD1-A, ZNF193-A, ZNF165-T). These define a restricted haplotype of approximately 500 Kb from now on designated as A-A-T haplotype. In contrast, the alleles associated with "high" CD8+ T-cell numbers are found in linkage disequilibrium in the new conserved haplotype defined by PGBD1-G, ZNF193-G, ZNF165-G. This restricted haplotype of approximately 500 Kb is from now on designated as G-G-G haplotype. The association of CD8+ T-lymphocyte numbers with the A-A-T haplotype (average CD8+T cells = 0.37 ± 0.17 × 10^6^/ml) and with the G-G-G haplotype (average CD8+ T cells = 0.55 ± 0.14 × 10^6^/ml) is more significant (p = 0.0108, one-way-ANOVA) than the association of CD8+ T cells with any of the markers alone.

#### 2.3. Analyses of combined conserved haplotypes

To further investigate the association found between the conserved haplotypes and CD8+ T-cell numbers, subjects were divided according to the combination of their two haplotypes. The conserved haplotype A-A-T was found in homozygosity in 50 subjects (89%) and the new conserved haplotype G-G-G was found always in heterozygosity with the ancestral in 6 subjects (11%).

A statistically significant result (p = 0.0092) was observed when association studies were performed between CD8+ T-lymphocyte numbers and subjects divided according to the combination of inherited haplotypes. Subjects carrying the two ancestral haplotypes (A-A-T × A-A-T) had significantly lower CD8+ T-cell counts (0.35 ± 0.17 × 10^6^/ml) and subjects carrying the combination of the new conserved haplotype with the ancestral haplotype (G-G-G × A-A-T) had significantly higher CD8+ T-cell counts (0.55 ± 0.14 × 10^6^/ml) (Table 2).

**Table 2:** Clinical, biochemical and immunological characterization of Portuguese HH patients, according to the combination of the two inherited conserved haplotypes*

	**A-A-T × A-A-T** **(n = 46)**	**G-G-G × A-A-T** **(n = 6)**	P**
**Age (years)**	46 ± 12	37 ± 8	*n*.*s*.
**Male/Female**	25/21	3/3	
**Transferrin saturation (%)**	87 ± 17	83 ± 13	*n*.*s*.
**Serum ferritin (ng/ml)**	1774 ± 1848	701 ± 1124	*n*.*s*.
**TBIS (g)**	7.58 ± 4.58	3.04 ± 1.52	*0.035*
**Symptomatic patients **	54% (25/46)	0% (0/6)	*0.04*

**Total CD8+ cells (× 10^6^/ml)**	0.35 ± 0.17	0.55 ± 0.14	*0.0092*
**Total CD4+ cells (× 10^6^/ml)**	0.96 ± 0.37	1.24 ± 0.40	*n*.*s*.
**Total lymphocytes (× 10^6^/ml)**	1.98 ± 0.57	2.65 ± 0.42	*0.0081*

* Conserved haplotypes are defined by SNP markers PGBD1-ZNF193-ZNF165 (see Haplotype definition in Material and Methods). Only one patient did not have any of these two conserved haplotypes and was not included in this analysis.

** Statistically significant differences between groups (*P*) were tested using Student's T-test (for mean values) or the Chi-square test with Yates correction (for categorical data). Parameters are expressed as mean ± standard deviation, except percentage of symptomatic patients. Estimation of total body iron stores (TBIS) was obtained in 37 patients with the haplotypes A-A-T × A-A-T and in 5 patients with the haplotypes G-G-G × A-A-T.

A statistically significant result (p = 0.0081) was also observed when association studies were performed between total lymphocyte numbers in subjects with two A-A-T haplotypes (1.98 ± 0.57 × 10^6^/ml) and subjects that carry the G-G-G haplotype (2.65 ± 0.42 × 10^6^/ml) (Table 2). No statistically significant differences were found in CD4+ T-lymphocyte numbers in subjects carrying or not the G-G-G haplotype (Table 2).

### 3. Implication of the conserved region defined by PGBD1-ZNF193-ZNF165 on the clinical expression of HH on Portuguese patients

The conserved region defined by the markers PGBD1-ZNF193-ZNF165 is part of the MHC class-I region previously shown to be associated with setting the level of CD8+ T-cell numbers [22]. This MHC class-I region was also shown to have an impact on the clinical expression of HH [22], therefore we tested the hypothesis that the conserved haplotypes described here (PGBD1-ZNF193-ZNF165) could also be associated with the phenotypic expression of HH. To investigate this hypothesis we analyzed the levels of TBIS and the presence of clinical manifestations of the disease in subjects divided according to the presence of the haplotypes A-A-T and G-G-G.

As shown in Table 2 the subjects heterozygous for the G-G-G haplotype (n = 6) have a much less severe expression of the disease than subjects homozygous for the A-A-T haplotype, as shown by the statistically significant lower levels of TBIS and the absence of clinical manifestations of the disease of the former. These differences were not reflected in the levels of TfSat and serum ferritin at diagnosis (Table 2).

### 4. Extension of the study done in Portuguese patients to a geographically different population of patients

Results described in this work showed that a conserved region around the microsatellite D6S105, defined by the markers PGBD1-ZNF193-ZNF165, is associated with CD8+ T-lymphocyte numbers and with the severity of the clinical expression in Portuguese HH patients. To extend this study to a different population of patients we studied a group of Canadian HH patients (n = 10). These patients were genotyped for the 5 SNP markers and total lymphocyte counts, and CD4+ and CD8+ subpopulations were also determined (see Material and Methods). A summary of data from these patients is shown in Table 3.

**Table 3:** Clinical and immunological characterization and inferred haplotypes present in a sample of 10 Canadian HH patients

		**Age**	**TfSat**	**Ferritin**	**TBIS**	**Associated**	**CD4+**	**CD8+**	**Haplotype 1**					**Haplotype 2**				
										
**ID**	**Gender**	(years)	(%)	(ng/ml)	(g)	** diseases**	(×10^6^/ml)		**ZSCAN12**	**PGBD1**	**ZNF193**	**ZNF165**	**ZNF184**	**ZSCAN12**	**PGBD1**	**ZNF193**	**ZNF165**	**ZNF184**
1	M	52	96	2210	5.0		0.95	0.26	**A**	**A**	**A**	**T**	**G**	**A**	**A**	**A**	**T**	**G**
2	M	54	97	1618	12.0		0.97	0.17	**A**	**A**	**A**	**T**	**G**	**A**	**A**	**A**	**T**	**G**
3	F	65	95	1002	7.5		0.30	0.14	**A**	**A**	**A**	**T**	**G**	**A**	**A**	**A**	**T**	**G**
4	F	63	n.a.	2550	28.5		0.66	0.21	**A**	**A**	**A**	**T**	**G**	G	**A**	**A**	**T**	T
5	M	43	n.a.	n.a.	25.0	Hepatitis C infection	0.80	0.43	**A**	**A**	**A**	**T**	**G**	**A**	**A**	**A**	**T**	**G**
6	F	61	60	500	2.0	Celiac disease	0.93	0.53	**A**	**A**	**A**	**T**	**G**	G	**A**	**A**	**T**	T
7	F	35	95	82	0.5	Hashimoto's thyroiditis	0.79	0.50	**A**	**A**	**A**	**T**	**G**	**A**	**A**	**A**	**T**	**G**
8	M	51	83	646	2.6		0.55	0.43	**A**	**A**	**A**	G	T	**A**	**A**	**A**	**T**	T

9	F	80	79	463	1.7	Small cell B lymphoma	0.64	0.40	**G**	**G**	**G**	**G**	G	G	**A**	**A**	**T**	T
10	M	39	45	695	2.1		1.01	0.58	**G**	**G**	**G**	**G**	G	**G**	**G**	**G**	**G**	T

M: male; F: female. Tfsat: transferrin saturation. n.a.: not available.

Alleles in bold represent conserved haplotype regions. Text in grey highlight subjects with associated diseases.

The majority of these patients (5/10) were homozygous for the extended haplotype defined by ZSCAN12-A, PGBD1-A, ZNF193-A, ZNF165-T, ZNF184-G, as found in the Portuguese HH patients (Patients #1, #2, #3, #5, #7). Moreover, some diversity was found in the markers ZSCAN12 and ZNF184 (patient #4, #6) but these patients were homozygous for the same highly conserved haplotype PGBD1-A, ZNF193-A, ZNF165-T (A-A-T haplotype) as found in the Portuguese HH patients. Interestingly, patients with the haplotype A-A-T (patients #1, #2, #3, #4, after exclusion of patients #5, #6 and #7 because of the associated diseases) were the ones with the lowest levels of CD8+ T lymphocytes and with the more severe forms of iron overload (Table 3).

In this group of patients it was identified for the first time one patient homozygous for the new conserved haplotype G-G-G (patient #10). This patient had the highest number of CD8+ T cells (0.58 × 10^6^/ml) and had a mild form of iron overload (TBIS = 2.1 g, Table 3).

One patient was found to be heterozygous for the new conserved haplotype G-G-G (patients #9). In this patient the number of CD8+ T cells and the clinical expression of HH could be confounded by the presence of a small cell B lymphoma.

### 5. Global analysis of the conserved haplotypes on CD8+ T-lymphocyte numbers and the clinical expression of HH

**Figure 2:**
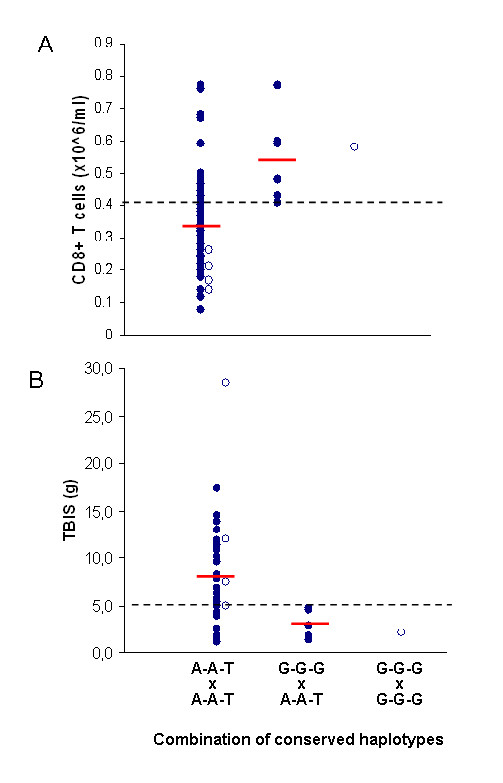
**Distribution of CD8+ T-lymphocyte numbers (A) and of total body iron stores (B) in HH patients.** Distribution of CD8+ T-lymphocyte numbers (A) and of total body iron stores (TBIS) (B) in all HH patients (Portuguese and Canadian), according to the combination of the conserved haplotypes (defined by SNP markers PGBD1-ZNF193-ZNF165), which divide subjects in three groups: patients homozygous for the ancestral A-A-T haplotype (n=50), patients heterozygous for the G-G-G haplotype (n=6) and homozygous for the G-G-G haplotype (n=1). Dashed lines represent: (A)-the median value of CD8+ T-cells (see Material and Methods); (B)-level of TBIS above which iron stores is considered severe. Average values are shown in red lines. Open circles represent Canadian patients.

When results from both populations were pooled, similar results were obtained. For the pooled analysis, patients were divided according to the combination of the inherited conserved haplotypes in three groups: patients homozygous for the ancestral A-A-T haplotype (n = 50), patients heterozygous for the G-G-G haplotype (n = 6) and homozygous for the G-G-G haplotype (n = 1). The results illustrated in Figure 2 clearly show that all patients carrying the new conserved haplotype G-G-G, either in heterozygosity or in homozygosity, have "high" CD8+ T-lymphocyte numbers and a mild expression of iron overload. All patients with severe iron overload (> 5 g) or with "low" CD8+ T-cell counts are homozygous for the haplotype A-A-T. Globally, the combination of the conserved haplotypes was significantly associated with both CD8+ T-lymphocyte numbers and the amount of iron overload measured by TBIS. Subjects homozygous for the A-A-T haplotype had statistically significant lower average values of CD8+ T-lymphocyte numbers (0.34 ± 0.17 × 10^6^/ml) in comparison to carriers (heterozygous or homozygous) for the G-G-G haplotype (0.55 ± 0.12 × 10^6^/ml) (p = 0.0022, Student T-test). Accordingly, statistically significant higher values of TBIS were found in A-A-T homozygous subjects (8.33 ± 5.64 g) in comparison with G-G-G carriers (2.88 ± 1.41 g) (p = 0.024, Student T-test).

## Discussion

This study followed previous observations that MHC class-I linked genetic markers associated with the setting of CD8+ T-cell numbers could be relevant modifiers of the clinical expression in HH. The study aimed to find new genetic markers that could be used as more reliable prognostic variables in the clinical management of HH patients and also to further narrow the region of interest to find a candidate gene involved in the setting of CD8+ T-lymphocyte numbers in humans.

The results confirmed that a highly conserved 500 Kb ancestral haplotype defined by the SNP markers PGBD1-A, ZNF193-A, ZNF165-T (A-A-T haplotype) marks the inheritance of "low" CD8+ T-lymphocyte numbers and predicts the development of a severe clinical expression of HH (in terms of iron overload and clinical manifestations). In a small proportion of patients, a new conserved haplotype defined by the SNP markers PGBD1-G, ZNF193-G, ZNF165-G (G-G-G haplotype) was found associated with both the inheritance of "high" CD8+ T-lymphocyte numbers and a milder clinical expression of HH. Very interestingly, the two conserved haplotypes defined in Portuguese patients, were also observed in another geographically different population of Canadian patients, also predicting CD8+ T-lymphocyte numbers and the severity of disease, supporting the view that they most probably are descendents of the same common north European ancestor as the Portuguese patients and therefore may carry the same genetic modifiers. When results of both populations were analyzed together (Figure 2) it was evident that the subjects homozygous for the A-A-T haplotype (n = 50) had the lowest average values of CD8+ T cells and highest average levels of TBIS. However there is still some heterogeneity in this group of patients, 28% (14/50) of them presenting with "high" CD8+ T cells and 29% (12/41) presenting a moderate or mild iron overload (TBIS < 5 g). In contrast, all subjects heterozygous for the new conserved haplotypes G-G-G (n = 6) and the only subject homozygous for the G-G-G haplotypes had CD8+ T-cell numbers > 0.41 × 10^6^/ml and levels of TBIS < 5 g. These results might be explained by the existence in this region of a genetic trait associated with CD8+ T-lymphocyte numbers modifying the phenotype of iron overload in HH. The fact that there is a high heterogeneity in CD8+ T-cell numbers and, consequently in TBIS, in the group of subjects carrying two copies of the A-A-T haplotype is not surprising. Vieira and co-workers [23] stated that individuals with genetically determined "low" CD8+ T cells may under some circumstances have "high" CD8+ T lymphocytes but the opposite is unexpected [23]. Subjects carrying the new conserved G-G-G haplotype were found in the present study only in a small proportion of cases. One may speculate, however, that they may eventually represent the tip of an iceberg where this haplotype could be common in the population of asymptomatic C282Y homozygous subjects. One limitation of this study is the fact that large numbers of asymptomatic or mildly affected hemochromatosis subjects were not available, as expected by the fact that they do not search for medical care. This study should be extended to test, in larger worldwide spread populations, if the G-G-G haplotype is also a marker of clinical expression of HH subjects detected in large population screening studies where a high proportion of subjects do not seem to express the disease [9-11].

Finally, the present results may have important implications in future strategies to narrow the region of interest to find a candidate gene involved in the settings of CD8+ T-lymphocyte numbers. Previous work from our group in C282Y homozygous patients [22] and in control subjects [23] had shown results suggesting that the gene(s) regulating CD8+ T lymphocytes is localized in the vicinity of the microsatellite D6S105. This was in accordance with previous work by Pratiwi et al. [24] that showed by an extended linkage disequilibrium analysis in the hemochromatosis gene region, two distinct peaks of association, namely a highly significant association at D6S2239, localized in close proximity to *HFE *(14 kilobases telomeric) and at D6S105 [24]. It the present work we use additional genetic markers in close proximity to the microsatellite D6S105 and found that this microsatellite marker itself was not associated with CD8+ T lymphocytes, but 3 other SNP markers defining a conserved 500 Kb haplotype and localized 400 Kb centromerically to D6S105, constitute the best markers described till now predicting CD8+ T-cell numbers and the phenotype of HH. It is very unlikely that any of these SNP markers contribute directly to the setting of CD8+ T lymphocytes, therefore further studies, with extended genetic markers and with increased numbers of subjects, are needed.

## Conclusion

These results may have important implications for approaching the question of the penetrance of the hemochromatosis gene in different world populations and also to further narrow the region of interest to find a candidate gene involved in the setting of CD8+ T-lymphocyte numbers in humans.

## Competing interests

The authors declare that they have no competing interests.

## Authors' contributions

EC and GP conceived and designed the study, diagnosed and treated the Portuguese hemochromatosis patients, compiled their clinical data, contribute to the interpretation of data and wrote the manuscript. Additionally, EC analyzed the data and performed the statistical analysis. CW diagnosed and treated the Canadian hemochromatosis patients. JV participated in the design of the study and contributed to the statistical analysis and its interpretation and to the writing of the manuscript. SK oversaw the performance and interpretation of most of the laboratory assays performed on the Canadian patients and assisted with correlations between clinical and laboratory findings. Additionally, CW and SK contribute to the interpretation and actively participate in the discussion of the results of the manuscript. CM performed the SNP genotyping of all patients. RL performed the T-cell immunophenotyping of the Portuguese patients. All authors read and approved the final manuscript.

## Pre-publication history

The pre-publication history for this paper can be accessed here:


